# Preceding propagation of turbulence pulses at avalanche events in a magnetically confined plasma

**DOI:** 10.1038/s41598-022-10499-z

**Published:** 2022-05-16

**Authors:** N. Kenmochi, K. Ida, T. Tokuzawa, R. Yasuhara, H. Funaba, H. Uehara, D. J. Den Hartog, I. Yamada, M. Yoshinuma, Y. Takemura, H. Igami

**Affiliations:** 1 National Institute for Fusion Science, Toki, Gifu 509-5292 Japan; 2 The Graduate University for Advanced Studies, SOKENDAI, Toki, Gifu 509-5292 Japan; 3 Wisconsin Plasma Physics Laboratory, University of Wisconsin-Madison, Madison, WI 53706 USA

**Keywords:** Magnetically confined plasmas, Plasma physics

## Abstract

The preceding propagation of turbulence pulses has been observed for the first time in heat avalanche events during the collapse of the electron internal transport barrier (e-ITB) in the Large Helical Device. The turbulence and heat pulses are generated near the foot of the e-ITB and propagate to the peripheral region within a much shorter time than the diffusion timescale. The propagation speed of the turbulence pulse is approximately 10 km/s, which is faster than that of the heat pulse propagating at a speed of 1.5 km/s. The heat pulse propagates at approximately the same speed as that in the theoretical prediction, whereas the turbulence pulse propagates one order of magnitude faster than that in the prediction, thereby providing important insights into the physics of non-local transport.

## Introduction

The lack of understanding of turbulent (anomalous) transport has been a core issue for more than half a century in magnetic fusion plasma research. Plasma transport cannot be explained using local models alone and requires an understanding of non-local transport, in which long-range responses are evident before diffusive transport effects reach these regions^1–4^. One mechanism potentially responsible for non-local transport is turbulence spreading, i.e., the propagation of fluctuation energy into different regions by nonlinear spectral transfer^5–12^. Turbulence spreading can decouple local turbulence intensity and therefore decouple turbulent diffusivity from the local gradient. Such an outcome results in the deviation from local transport models. In the literature, turbulence spreading is utilized to explain various non-local transport phenomena^6,7,13–16^. The formation of avalanches is another phenomenon closely related to non-local transport. Avalanches occur in the dynamics of a marginally stable system and can play a role in non-local transport events^17^. Although some experimental results have been reported^18–22^, the observations of turbulence spreading and avalanches require diagnostics with high sensitivity and spatio-temporal resolution, and reports on such experimental observations are currently limited. Here we report the propagation characteristics of the avalanches and turbulence pulses generated during the collapse of an electron internal transport barrier (e-ITB) using the advanced diagnostics of the Large Helical Device (LHD). The LHD is suitable for research based on the observation of avalanche and turbulence spreading because it has advanced diagnostic capabilities, and its magnetic field structure can be maintained even when the e-ITB collapses. In this study, strong pressure gradients are generated by the formation of magnetic islands inside the e-ITB plasma, which induce instabilities and generate observable turbulence that is larger than the background turbulence. Such large turbulence enables us to observe the propagation of the turbulence pulses. This method can artificially induce avalanche and turbulence spreading caused by the collapse of the e-ITB and is beneficial for understanding the physics of avalanche and turbulence spreading.

## Results

### Experimental set-up

**Figure 1:**
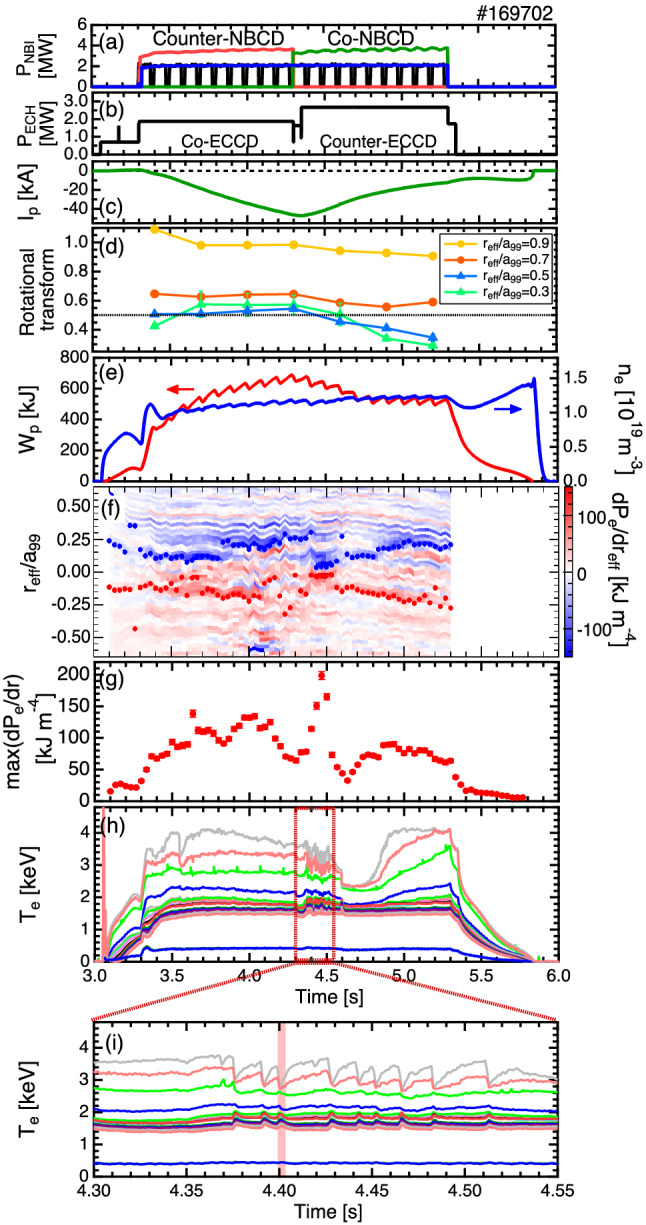
Time evolutions of the (**a**) heating power of the NBI, (**b**) that of the ECRH, (**c**) plasma current, $I_p$, (**d**) rotational transform for each radial position, (**e**) stored energy in the plasma, $W_p$, and line-averaged electron density, ${\bar{n}}_{e}$, (**f**) profile of the electron pressure gradient, (**g**) maximum value of the electron pressure gradient, (**h**) electron temperature measured by ECE, and (**i**) electron temperature during the minor collapse of the e-ITB. The black horizontal line in Fig. 1d denotes a rotational transform value of 0.5. In Fig. 1f, the radial positions of the maximum pressure gradient are indicated using blue and red dots.

**Figure 2:**
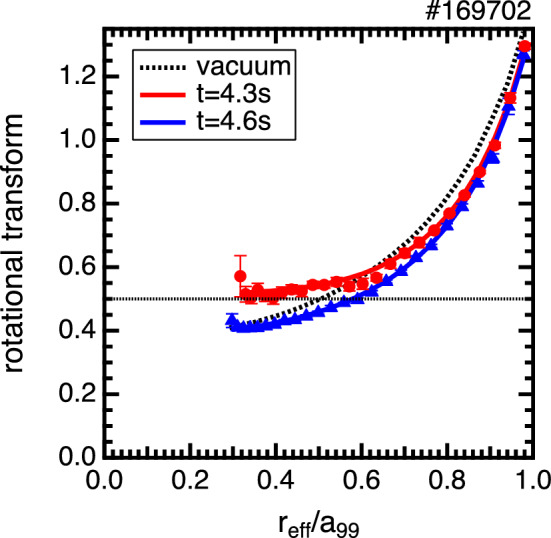
Rotational transform profiles before ($t=4.3$ s, red) and after ($t=4.6$ s, blue) the change in the direction of current drive. Additionally, the black dotted line shows the rotational transform profile in a vacuum magnetic field. The black horizontal line denotes a rotational transform value of 0.5.

The experiments are conducted in the LHD, which is a heliotron-type device for the magnetic confinement of high-temperature plasmas^23^. The LHD possesses three tangential neutral beams, out of which two beams are used to drive the plasma current in a direction parallel (co-injection) or antiparallel (counter-injection) to the equivalent plasma current, and one beam is always used as a probe beam of the motional Stark effect (MSE) spectroscopy^24^. In this experiment, the electron cyclotron current drive is used to control the rotational transform of the core region while maintaining the e-ITB^25,26^. Figure 1 shows the time evolutions of the heating power of the neutral beam injection (NBI) and electron cyclotron resonant heating (ECRH), plasma current, rotational transform, plasma stored energy, line-averaged electron density, profile of the electron pressure gradient, and electron temperature. The electron temperature ($T_e$) is measured using an electron cyclotron emission (ECE) radiometer^27^. To form a magnetic island in the e-ITB plasma, the rotational transform profile is dynamically changed by switching the current direction driven by tangential NBI and oblique electron cyclotron wave injection in the core region. Figure 1d shows the time evolutions of the rotational transforms for each radial position ($r_{\mathrm {eff}}/a_{99} = 0.3$, 0.5, 0.7, and 0.9), and Fig. 2 shows the rotational transform profiles before and after the change in the current drive direction. The rotational transform around the center dynamically changes with time to 0.6 from 0.4 before the change in the current drive direction and to 0.4 from 0.6 after the change in the current drive direction. The saddle loop and MSE measurements show that a static $n/m = 1/2$ magnetic island is formed after changing the driving direction of the plasma current.

### Minor collapse of the e-ITB

The minor collapse of the e-ITB is repeatedly observed after changing the driving direction of the plasma current. Because the NBI is modulated at 10 Hz for the MSE measurement, the stored energy and line-averaged electron density are perturbed. Since the fluctuation ranges are small, they are considered to have no effect on the minor collapse of the e-ITB. During the minor collapse of the e-ITB, the $T_e$ in the center region decreases, whereas the $T_e$ around the foot of the e-ITB increases. In the peripheral region, the $T_e$ increases rapidly and subsequently decreases slowly, unlike that in the central region.

**Figure 3:**
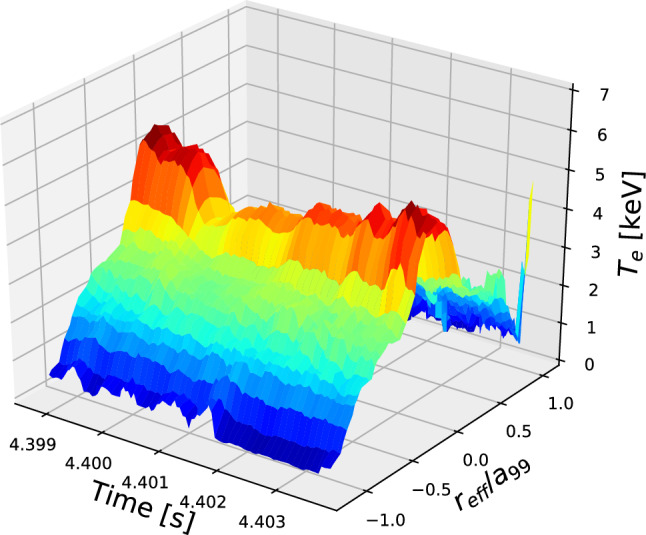
Time evolutions of the electron temperature profiles during the minor collapse of the e-ITB measured using the Thomson scattering with high-time resolution of 20 kHz repetition rate. Here, $r_{\mathrm {eff}}/a_{99}$ is the normalized coordinate, $r_{\mathrm {eff}}$ is the effective minor radius, and $a_{99}$ is the minor radius of the flux surface enclosing 99% of the stored energy.

To investigate the change in the $T_e$ observed in the ECE in detail, we apply a newly developed Thomson scattering measurement with high-time resolution^28^. Figure 3 shows the time evolution of the electron temperature profile during the minor collapse event. The high-time resolution Thomson scattering measurement is conducted using 20 kHz sampling for $\sim$ 5 ms, and the measured time is shown using red hatching in Fig. 1i. The detailed profile change during the minor collapse of the e-ITB is revealed in Fig. 3. The central $T_e$ decreases rapidly, whereas the peripheral $T_e$ increases rapidly. After the collapse of the e-ITB, the $T_e$ of the center slowly increases, and the e-ITB is restored. Furthermore, high time and space-resolved measurements reveal that the electron temperature near the magnetic axis increases with oscillation during the re-formation of the e-ITB. As shown in Fig. 1f,g, by changing the current direction, a strong pressure gradient of more than 150 ${\text {kJ m}}^{-4}$ is formed at the foot of the e-ITB, close to the center, owing to the formation of a magnetic island. When the pressure gradient reaches this value, the e-ITB collapses. After the collapse, the temperature gradient increases again, thereby leading to a subsequent collapse. The $T_e$ decreases in the region inside the e-ITB with $r_{\mathrm {eff}}/a_{99} < 0.3$, whereas it increases outside the e-ITB (see Fig. 7a). More specifically, when the e-ITB collapses, the heat inside the e-ITB propagates to the outside in the form of heat pulses.

### Observation of the turbulence pulse just before the heat pulse

**Figure 4:**
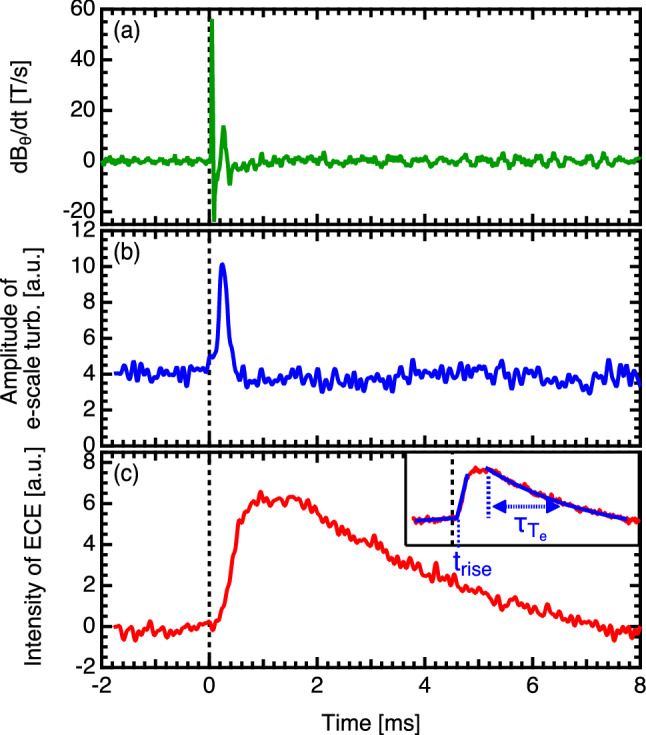
Time evolutions of the (**a**) magnetic fluctuation, (**b**) electron scale turbulence, and (**c**) electron temperature measured using a magnetic probe, W-band back-scattering measurement, and ECE measurement, respectively.

Increases in the magnetic fluctuations and electron scale turbulence are observed in synchronizing with the minor collapse of the e-ITB. Figure 4 shows the time evolution of the magnetic fluctuation, electron scale turbulence at $r_{\mathrm {eff}}/a_{99} = 0.46$, and electron temperature at $r_{\mathrm {eff}}/a_{99} = 0.47$ measured using a magnetic probe, a 90 GHz W-band millimeter-wave back-scattering measurement^29^, and the ECE measurement, respectively. The W-band back-scattering diagnostics measured the electron-scale turbulence that had a high wavenumber of $k_\perp \rho _s \sim 40$ and $k_\perp \sim 40\, {\mathrm {cm}}^{-1}$, where $k_\perp$ is the fluctuation wavenumber perpendicular to the magnetic field and $\rho _s$ is the ion gyro radius at the electron temperature. Here, the sum of the fluctuation intensities in the range of 20–200 kHz for the back-scattering measurement is shown in the figure. The profiles of the turbulences are measured by scanning the direction of the receiving antenna shot by shot. To improve the signal-to-noise ratio, the conditional averaging technique with respect to the time of a sharp increase of $T_e$ measured by the ECE is applied to investigate the amplitude of the density fluctuations and electron temperature changes during the collapse event with a time resolution of $1\times 10^{-6}$ s and $1\times 10^{-5}$ s, respectively. The time resolutions of both measurement were high enough to discuss the dynamics of turbulence during the collapse. In Fig. 4, the rise time of the turbulence pulse at the position $r_{\mathrm {eff}}/a_{99}=0.21$, where the earliest turbulence pulse is observed (see Fig. 6), is plotted as $t=0$ s. The magnetic fluctuation and the electron-scale turbulence rapidly increases just before the minor collapse of the e-ITB occurs. Notably, since the back-scattering measurement is performed in O-mode, there is no direct effect of the magnetic field fluctuations on the back-scattering measurement, unlike that in the case of the X-mode. In addition, the time differences between the back-scattering measurement signals at different spatial positions suggest that the magnetic field fluctuation do not affect this measurement; instead, these fluctuations indicate the presence of density fluctuations. When the pressure gradient increases to a threshold value, the MHD instability coincides with the enhanced electron scale turbulence and a minor collapse occurres. The $T_e$ and $T_e$ gradient in the central region increases again, and the e-ITB recovers. The intensity of the magnetic field fluctuation and electron scale turbulence increases rapidly for approximately 300 μs, thereby suggesting the existence of a pulse shape with a line-of-sight of the back-scattering measurement of approximately 0.1 m, whereas the $T_e$ increases and decreases throughout at approximately 10 ms. The spatio-temporal characteristics of the electron-scale turbulence suggest that it possesses a pulse shape (i.e., turbulence pulse).

### Self-similar characteristics of the collapse events

**Figure 5:**
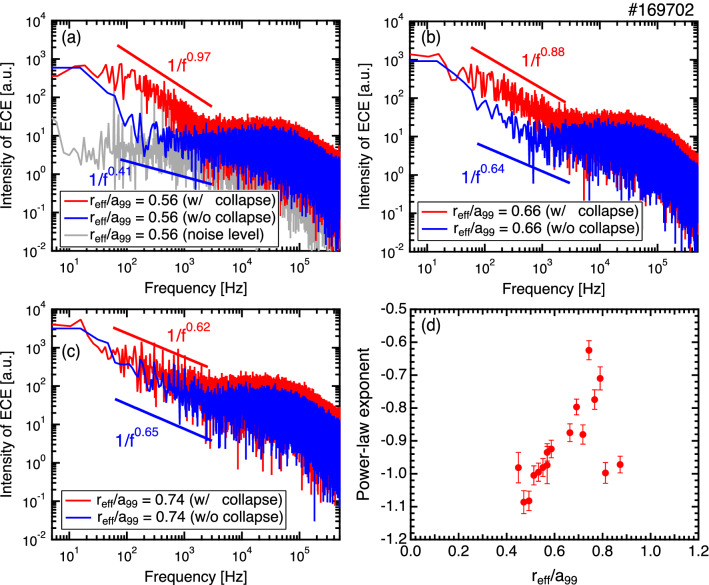
Frequency spectrum of the ECE signal with (red, $t=4.366$–4.527 s) and without (blue, $t=4.300$–4.366 s) the minor collapse events at $r_{\mathrm {eff}}/a_{99} =$ (**a**) 0.56, (**b**) 0.66, and (**c**) 0.74. (**d**) Power-law exponents during the minor collapse events derived from the frequency spectrum between 70–3000 Hz for different spatial channels of the ECE signals.

Avalanche events are well known for displaying self-similar behavior. The self-similar characteristic of a fluctuation can be identified from the behavior of the frequency spectrum with a power-law^2,30^. The power-law tail on the frequency spectrum is an indication of the increased importance of rare large events. Another indication is the Hurst parameter *H* that directly characterizes the behavior of the autocorrelation at large lag times^31^. Figure 5a–c show the frequency spectra of the ECE intensities with ($t=4.366$–4.527 s) and without ($t=4.300$–4.366 s) the occurrence of the minor collapse events at $r_{\mathrm {eff}}/a_{99} = 0.56$, 0.66, and 0.74, respectively. The power-law exponents calculated for the range of 70–3000 Hz are also shown in these figures. Here, when the spectrum is expressed using $S(f) \propto f^{\alpha }$, $\alpha$ is called the power-law exponent. In the range of 70–3000 Hz, the spectra of the ECE signals measured around the foot of the e-ITB during the minor collapse events show a $\sim 1/f$ dependence (e.g. $1/f^{0.97}$ for $r_{\mathrm {eff}}/a_{99} = 0.56$), whereas the power-law exponents without the collapse events are higher than $-\,0.7$. Here, the spectrum in the absence of the plasma, which indicates the noise level of the measurement, is also shown in Fig. 5a, where the power-law exponent is approximately $-\,0.02$. Figure 5d displays the power-law exponents at each measurement position in the 70–3000 Hz range during the collapse events. The value of the power-law exponent is higher (lower spectral slope) for signals at outward measurement positions away from the foot of the e-ITB. This result indicates that the avalanche generated near the foot of the e-ITB propagates outwards. More specifically, a strong self-similarity is observed around the foot of the e-ITB, which is the source of the avalanche event, whereas the self-similarity is weakened in the peripheral region where the avalanche-generated heat pulse is observed. The intensities of the heat pulses are small and may not be observed owing to the noise in the measurement; however, the scale length is determined to be approximately 0.3 in $r_{\mathrm {eff}}/a_{99}$ if we assume that the range in which the power-law exponents of $-\,1.0 \pm 0.1$ is the scale length of avalanche.

The Hurst parameter between the time lag of $1 \times 10^{-6}$ s to $1 \times 10^{-2}$ s is $H \sim 0.96$ at $r_{\mathrm {eff}}/a_{99} = 0.56$. The time evolution of $T_e$ shows intermittent and large radial-scale events with a 1/*f* spectrum and strong evidence from the Hurst parameter of a high recurrence rate for large events^32^. Additionally, radial propagation is clearly observed. From the above discussion, the time evolution of $T_e$ during the collapse of the e-ITB has all of the qualitative characteristics expected from avalanches.

**Figure 6:**
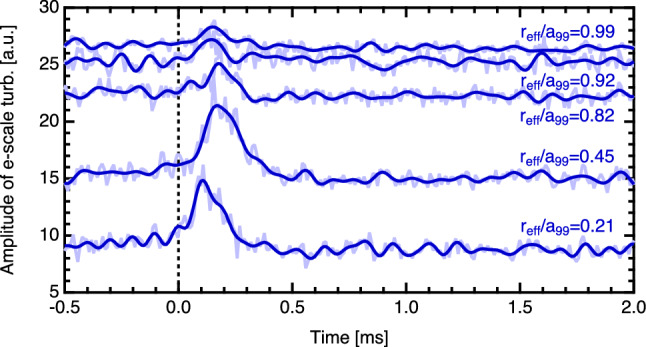
Time evolutions of the electron scale turbulences for different radial positions obtained from the W-band back-scattering measurement. The light-blue lines show the raw data, and dark-blue lines show the data after low-pass processing below 20 kHz.

### Propagation of the turbulence pulses preceding the heat pulses

**Figure 7:**
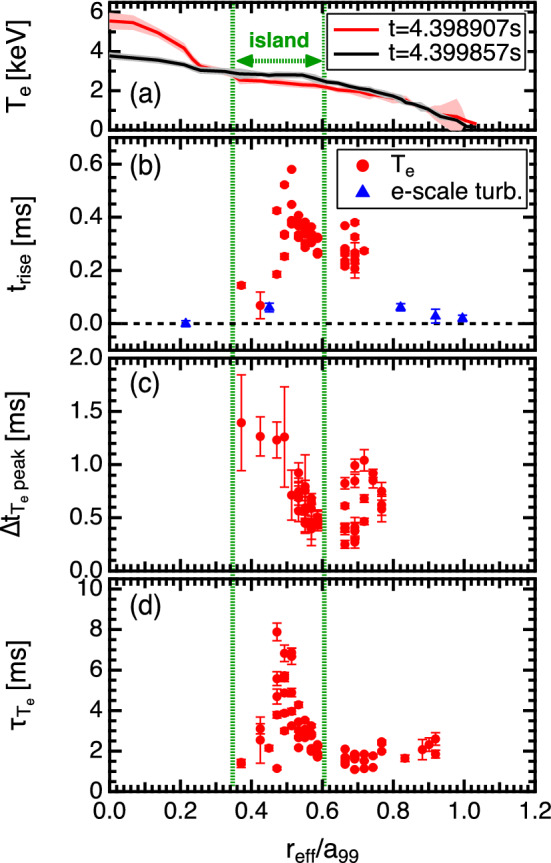
Profiles of the (**a**) electron temperature (red) before and (black) after the collapse of the e-ITB, (**b**) rise time of the electron temperature and electron scale turbulence, (**c**) interval between the rise time and peak time of the electron temperature, and the (**d**) decay time of the electron temperature.

The time evolutions of the electron scale turbulences measured at different spatial locations are shown in Fig. 6. Here, the rise time of the turbulence pulse at $r_{\mathrm {eff}}/a_{99}=0.21$ is defined as $t=0$ s. From this figure, the turbulence pulse can be seen to propagate outward, and the steepness of the pulse rise slows down as it moves outward. Figure 7 shows the profiles of the electron temperatures before and after the collapse of the e-ITB, rise times $t_{\mathrm {rise}}$ of the electron temperature and electron scale turbulence, the interval between the $t_{\mathrm {rise}}$ and the peak time of the electron temperature, and decay time of electron temperature. In this figure the flat shape of the electron temperature and characteristics of the heat pulse propagation allowed us to determine the region of magnetic island, $0.35<r_{\mathrm {eff}}/a_{99}<0.60$, as shown by the green dotted line. The thermal and turbulence pulses are analyzed only for the signals with intensities that can be distinguished from the noise; therefore, the plots are not shown for the measurement locations where no significant signal change is observed. The $t_{\mathrm {rise}}$ of the $T_e$ is determined by fitting the ECE signal, using a Leaky ReLU-like function (see Fig. 4c) as follows:

1 $$\begin{aligned} f(t) = {\left\{ \begin{array}{ll} a\,t &{}\quad \hbox { if }\ t<t_{\mathrm {rise}}\\ b\,t &{} \quad \hbox { if }\ t \ge t_{\mathrm {rise}} \end{array}\right. } \end{aligned}$$

Here, *t* is the time and *a* and *b* are slopes before and after the $t_{\mathrm {rise}}$, respectively. In this figure, $t=0$ s is also defined as the $t_{\mathrm {rise}}$ of the turbulence pulse at $r_{\mathrm {eff}}/a_{99}=0.21$. The interval between the $t_{\mathrm {rise}}$ and peak time of the electron temperature is defined as $\Delta t_{T_e {\mathrm {peak}}}$. The decay time of the electron temperature ($\tau _{T_e}$) is estimated by fitting the curve of the ECE signal using an exponential function (see Fig. 4c) as follows:

2 $$\begin{aligned} f(t) = A\exp \left( -\frac{t-t_0}{\tau _{T_e}}\right) + y_0 \end{aligned}$$

Here, *A*, $t_0$, and $y_0$ denote the ECE intensity, offset time, and offset of the ECE signal, respectively. The $t_{\mathrm {rise}}$ of electron temperature is large around $r_{\mathrm {eff}}/a_{99}=0.5$, where the magnetic island is thought to exist, and decreases near the edge of the island, reflecting the fact that the propagation speed of the heat pulse is slower inside the island than at the edge. The effect of the magnetic island is also seen in the $\Delta t_{T_e {\mathrm {peak}}}$ and $\tau _{T_e}$. The rate of increase of $T_e$ is small inside the magnetic island and large near the edge of the magnetic island as plotted in Fig. 7c. The Fig. 7d shows that the rate of decrease of $T_e$ is slow in the magnetic island. These results are consistent with the reported results that indicate that the heat pulses struggle to penetrate the magnetic island and the propagation speed of the heat pulses in the island is low^33^, which indicates that the heat pulse is frozen to the magnetic field lines. This is also evidence of the formation of magnetic islands in the region in which $0.35<r_{\mathrm {eff}}/a_{99}<0.60$. Alternatively, the turbulence is not a magnetic flux surface function and is unrestricted by the fine magnetic island structure; therefore, it can propagate much faster than that of the heat pulse even in magnetic islands.

During the minor collapse, the turbulence and heat pulses propagated from the foot of the e-ITB to the peripheral region, thereby suggesting turbulences generated near the foot and/or the MHD instability triggered the propagation of heat pulses. The turbulence is observed even inside the magnetic island where the electron temperature is flattened. Since turbulence cannot be driven without a gradient, the turbulence observed inside the island is most likely to be spread turbulence. As shown in Fig. 7b, the electron scale turbulence propagates from $r_{\mathrm {eff}}/a_{99}=0.2$ to the peripheral region in approximately 50 μs, whereas the heat pulse propagates in approximately 200–600 μs. More specifically, the turbulence pulse with a speed of 10 km/s precedes the heat pulse propagating at 1.5 km/s. The propagation timescales of the turbulence and heat pulses are much shorter than the diffusive scale ($\sim$ several ms), and their spatial propagation scales are long ($\sim$ minor radius of the device) which are much longer than the ion gyro radius, thereby showing the characteristics of non-local transport.

## Discussion

Turbulence spreading is often modeled theoretically using a reaction-diffusion equation (Fisher-KPP equation) and the models suggest that a turbulence front propagates ballistically at a speed of $V \sim (\gamma D)^{1/2}$, where $\gamma$ is the local turbulence growth rate and *D* is the local mean turbulent diffusivity^34,35^. By taking $\gamma \sim v_{\mathrm {Thi}}/a$ and *D* as gyro-Bohm (GB) diffusivity $D_{\mathrm {GB}} \sim \rho _s^2 v_{\mathrm {Thi}}/a$, where $v_{\mathrm {Thi}}$ is the ion thermal velocity, *a* is the minor radius, and $\rho _s$ is the ion gyro radius, the propagation speed of the front is typically estimated to be $V \sim 1$ km/s for this experiment. The propagation speed of the heat pulse observed in this experiment is approximately equal to the theoretical prediction; however, the turbulence pulse propagates several times to one order of magnitude faster. Although the simultaneous propagation of the turbulence and heat pulses has been shown in some cases^20^, the observed phenomenon in the LHD is considered to follow a different mechanism. The heat pulse propagation experiment using a modulated ECRH in the DIII-D tokamak demonstrated that the density fluctuation propagated faster than the heat pulse near the O-point of the magnetic island^22^. As reported by the DIII-D, there are phenomena where turbulence and heat pulses propagate separately. In the DIII-D, the other experiments also demonstrated that the avalanche events with a short time scale propagated faster than those with a long time scale^19^. In our experiment, the turbulence pulse has a much smaller pulse width than that of the heat pulse, which is qualitatively consistent with the experimental results. Our experimental results show that the avalanche phenomena in this study may not be locally driven and is possibly unrelated to the local gradient. This indicates that the turbulence can propagate separately from the heat pulse. The difference in the waveforms of the turbulence and heat pulse also indicates that these propagate separately. Our experimental results suggest that the propagation of the turbulence and heat flux can have various speeds depending on the time scale or field conditions and is an example of a phenomenon that deviates from the model dealing with single-speed propagation. In conclusion, the propagation characteristics of the preceding turbulence pulses during avalanche events are presented for the first time using advanced diagnostics and a conditional averaging technique. The turbulence and heat pulses propagate faster than the diffusion time scale; however, the turbulence pulse is approximately one order of magnitude faster than that of the heat flux. This study demonstrates the existence of turbulence and heat propagation phenomena that cannot be explained within the scope of the existing models of avalanche and turbulent spreading; however, this study can provide essential insights into the physical mechanisms of non-local transport.

## Methods

### Large helical device

The LHD is a heliotron-type device, which is a toroidal device with non-axisymmetric coils that provide an average poloidal field for the magnetic confinement of high-temperature plasmas with a major radius at the magnetic axis of $R_{\mathrm {ax}} = 3.6$ m, an averaged minor radius of $a=0.6$ m, and a magnetic field at the axis of up to 2.75 T^23^. In this experiment, the line-averaged density of the plasma is approximately constant at ${\bar{n}}_{e}=1.0\times 10^{19}\, {\mathrm {m}}^{-3}$ and the e-ITB is maintained from the beginning of the discharge. The target plasma is produced using an ECRH of 1 MW. The LHD is equipped with ECRHs with frequencies of 77 and 154 GHz and three tangential neutral beams (NBs) in opposite injection directions (two counterclockwise and one clockwise) and beam energy in the range of 160–180 keV and two perpendicular beams with beam energy in the range of 40–50 keV. The electron cyclotron current drive (ECCD) is used to control the rotational transform of the core region while maintaining the e-ITB^25,26^. The NBs are also used to control the rotational transform profile with the toroidal current driven by the NB. Here, the rotational transform  is the inverse of the safety factor  in tokamaks and is defined as , where *R* and *r* are major and minor radii, respectively, and $B_\theta$ and $B_\phi$ are the poloidal and toroidal magnetic fields, respectively.

### EC and NB current drive

ECCD and NB current drive (NBCD) are useful tools to drive the toroidal current. The total plasma current driven by the EC and NB is 50 kA, which is only $3\%$ of the equivalent plasma current (1.8 MA) that provides the poloidal field produced by the external coil current. The ECCD plays an important role in controlling the rotational transform profile in the core region in this experiment owing to its ability to control the spatially localized current profile. The inductive current in the direction opposite to the toroidal current owing to the NBCD in the core region also plays an important role in this experiment. To form a magnetic island in the e-ITB plasma, the rotational transform profile is dynamically changed by switching the current direction driven by the tangential NB and oblique EC wave injections in the core region. The saddle loop measurements show that a static $n/m=1/2$ magnetic island is formed after changing the driving direction of the plasma current. Notably, the saddle loop measures the radial magnetic field, and there are two sets of 12 saddle coils arranged in the poloidal direction 180$^{\circ }$ apart in the toroidal direction^36^. The number of identified poloidal modes are up to six, and the number of identified toroidal modes are even odd.

### High time-resolution Thomson scattering measurement

High-time resolution Thomson scattering (TS) system can measure the electron temperature and density up to 20 kHz with high-spatial resolution (70 spatial points) in the LHD^28,37^. The main technology of this system consists of a high repetition rate laser system and an analog to digital converter (ADC) for the signal from the polychromators. The high repetition rate laser system was realized using the burst mode operation of a flash lamp pumped Nd:YAG laser source^38,39^. This provides over 100 pulses of 20 kHz laser output with a laser energy of 1 J and pulse duration of 20 ns. The ADCs (TechnoAP APV85G32L) can store the high repetition rate TS signals up to 20 kHz as a waveform. And currently, around 70 spatial channels are connected to the new ADCs for measuring the TS system.

### High-k back-scattering measurement

A 90 GHz W-band millimeter-wave back-scattering system measures electron scale turbulence ($k_\perp \rho _s \sim 40$)^29^. To accurately observe the intensity of the scattered signal, which is proportional to the square of the electron density change in the plasma, with high spatial resolution, a collinear focusing optical antenna with a metallic lens is installed in the LHD vacuum vessel, and the beam diameter is kept below 40 mm. The estimated size of the scattering volume is $\sim$ 105 mm at the edge and 135 mm at the core, respectively, which is equivalent to a length of about 0.1–0.2 in $r_{\mathrm {eff}}/a_{99}$. This collinear antenna allows the observation position to be changed from the plasma core to the edge by a remote steering mechanism. The millimeter-wave heterodyne detection circuit also includes a modulation function to identify noise components in the scattered signal that are caused by cyclotron radiation of electrons emitted from the plasma.

## Data Availability

The raw data were generated at the LHD facility. The automatic Integrated Data Analysis software and the analyzed data are available from the LHD data repository located at https://www-lhd.nifs.ac.jp/pub/Repository_en.html.
